# A new biomechanical FE model for blunt thoracic impact

**DOI:** 10.3389/fbioe.2023.1152508

**Published:** 2023-03-22

**Authors:** Martin Chaufer, Rémi Delille, Benjamin Bourel, Christophe Marechal, Franck Lauro, Olivier Mauzac, Sebastien Roth

**Affiliations:** ^1^ Interdisciplinary Laboratory Carnot of Bourgogne-Site UTBM, UMR 6303, CNRS / Université Bourgogne Franche-Comté (UBFC), Belfort, France; ^2^ Université Polytechnique Hauts-de-France, CNRS, UMR 8201 – LAMIH – Laboratoire d’Automatique de Mécanique et d’Informatique Industrielles et Humaines, Valenciennes, France; ^3^ Insa Hauts-de-France, Valenciennes, France; ^4^ French Ministry of Interior, CREL/SAILMI, Paris, France

**Keywords:** blunt impacts, finite element model, human thorax, numerical twin, non-penetrating ballistics, body armor blunt trauma (BABT)

## Abstract

In the field of biomechanics, numerical procedures can be used to understand complex phenomena that cannot be analyzed with experimental setups. The use of experimental data from human cadavers can present ethical issues that can be avoided by utilizing biofidelic models. Biofidelic models have been shown to have far-reaching benefits, particularly in evaluating the effectiveness of protective devices such as body armors. For instance, numerical twins coupled with a biomechanical model can be used to assess the efficacy of protective devices against intense external forces. Similarly, the use of human body surrogates in experimental studies has allowed for biomechanical studies, as demonstrated by the development of crash test dummies that are commonly used in automotive testing. This study proposes using numerical procedures and simplifying the structure of an existing biofidelic FE model of the human thorax as a preliminary step in building a physical surrogate. A reverse engineering method was used to ensure the use of manufacturable materials, which resulted in a FE model called SurHUByx FEM (Surrogate HUByx Finite Element Model, with HUByx being the original thorax FE model developed previously). This new simplified model was validated against existing experimental data on cadavers in the context of ballistic impact. SurHUByx FEM, with its new material properties of manufacturable materials, demonstrated consistent behavior with the corresponding biomechanical corridors derived from these experiments. The validation process of this new simplified FE model yielded satisfactory results and is the first step towards the development of its physical twin using manufacturable materials.

## 1 Introduction

In recent decades, there has been a growing interest in the development of protective devices in various fields. More recently, researchers have focused on ballistic protection assessment. The development of such equipment requires an understanding of the human response to ballistic threats. To do so, two methods exist: the first consists of conducting experiments on instrumented humans or cadavers to collect data. These experiments are governed by strict ethical rules that make them complex to conduct. The second method is to mimic the response of the human body by using surrogates. Currently, only the use of Roma Plastilina Clay No. 1 is standardized by the NIJ (National Institute of Justice, 2008). However, other materials are commonly used to mimic human tissue, such as 10% or 20% ballistic gelatin, Perma-gel, ballistic soap, or synthetic gel based on Styrene-Ethylene-Butylene-Styrene (SEBS) (Read et al., 2022). In addition to the materials, human anthropometry should also be taken into consideration; anthropomorphic human surrogates have also been developed in the literature, such as in the studies on Ausman (Bass et al., 2006), SSO (Skin Skeleton Organs) (Wenmin et al., 2020), MHS (Modular Human Surrogate) (Sedberry and Foley, 2019), HSTM (Human Surrogate Torso Model) (Biermann et al., 2006), and BTTR (Blunt Trauma Torso Rig) (Bolduc and Anctil, 2010).

Recently, with the increase in computational power, researchers have developed numerous numerical surrogates. Shen et al. developed the Jaycor FE model (Shen et al., 2003) and the SSFEM (Subject-Specific Finite Element Model) (Shen et al., 2008), which is used for blunt trauma studies. Robert et al. developed the HTFEM (Human Torso Finite Element Model) (Roberts et al., 2005a; Roberts et al., 2007a; Merkle et al., 2008). The SHTIM (Surrogate Human Thorax for Impact Model) was developed by Nsiampa et al. (Nsiampa, 2011) to simulate less-lethal impacts. Kang et al. developed a thorax FE model equipped with soft armor (Kang et al., 2012). The ATBM (Advanced Total Body Model) was developed by Laurel et al. (Laurel and Eugene, 2018) in order to estimate the risk of injuries from kinetic energy weapons. Tang et al. developed a human torso to study the blunt trauma behind armor (Tang et al., 2019). Roth et al. developed the HUByx (Hermaphrodite universal Body YX) (Roth et al., 2013), while Cronin et al. developed the WALT (Waterloo Thorax Model), an FE model for blunt ballistic evaluation (Cronin et al., 2021). These types of numerical surrogates help understand complex phenomena that cannot be analyzed in experimental facilities. The use of such models has already proven their efficiency in the assessment of protective devices such as body armor.

Once created, surrogates (either numerical or physical ones) were compared with experimental data to ensure their biofidelity. Some numerical models such as HUByx (Roth et al., 2013; Bracq et al., 2019a), SHTIM (Nsiampa, 2011), or WALT (Cronin et al., 2021) are consistent with biomechanical corridors and/or different field impact cases. To the authors’ knowledge, only a few physical surrogates in the open literature were consistent with ballistic biomechanical corridors, such as BTTR (Bolduc and Anctil, 2010). Robert et al. developed both numerical and physical twin surrogates (Roberts et al., 2005b; Roberts et al., 2007b). Some similarities were found between these two models (HSTM and HTFEM). However, since the original numerical model was not considered biofidelic, the physical model was not regarded as biofidelic either. Therefore, in order to develop a biofidelic physical surrogate of the human thorax, the authors proposed a reverse engineering method: using a biofidelic numerical model as a basis to develop a biofidelic physical surrogate.

Following this method, and to be manufacturable, the structure of the initial FE model must be simplified. Since the material laws implemented in the initial FE model were extracted from humans material properties, materials available in the industry having similar properties as the ones implemented in the simplified FE model have to be selected to build the physical surrogate. Finally, a physical surrogate was built using the selected manufacturable materials and shapes of the simplified FE model.

Consequently, this study proposes to simplify the structure of an existing biofidelic FE model of a 50th percentile human thorax (the HUByx model) and to implement material laws for manufacturable materials. Once validated, this new simplified FE model called SurHUByx FEM (for Surrogate Hermaphrodite universal Body YX Finite Element Model), which is also a 50th percentile, will be the basis for building its physical twin and will be used for protection assessment.

## 2 Materials and methods

The HUByx FE model (Roth et al., 2013; Chaufer et al., 2021) is used as a reference and starting point. The model is then simplified to create a new FE model called SurHUByx FEM. This new model is designed to have a manufacturable structure and uses the material laws of manufacturable materials available in the industry. These two factors are crucial for the development of the physical twin called SurHUByx. To achieve this, the cortical and trabecular properties of both bones and cartilage were homogenized. Once the homogenized properties were computed, several manufacturable materials were tested to find the materials with the closest mechanical properties. Then they were implemented in the SurHUByx FEM. Then, comparisons were made with the literature to estimate the SurHUByx FEM anthropometry. Finally, well-known tests were replicated on the SurHUByx FEM to compare its response with biomechanical corridors as a validation.

**FIGURE 1:**
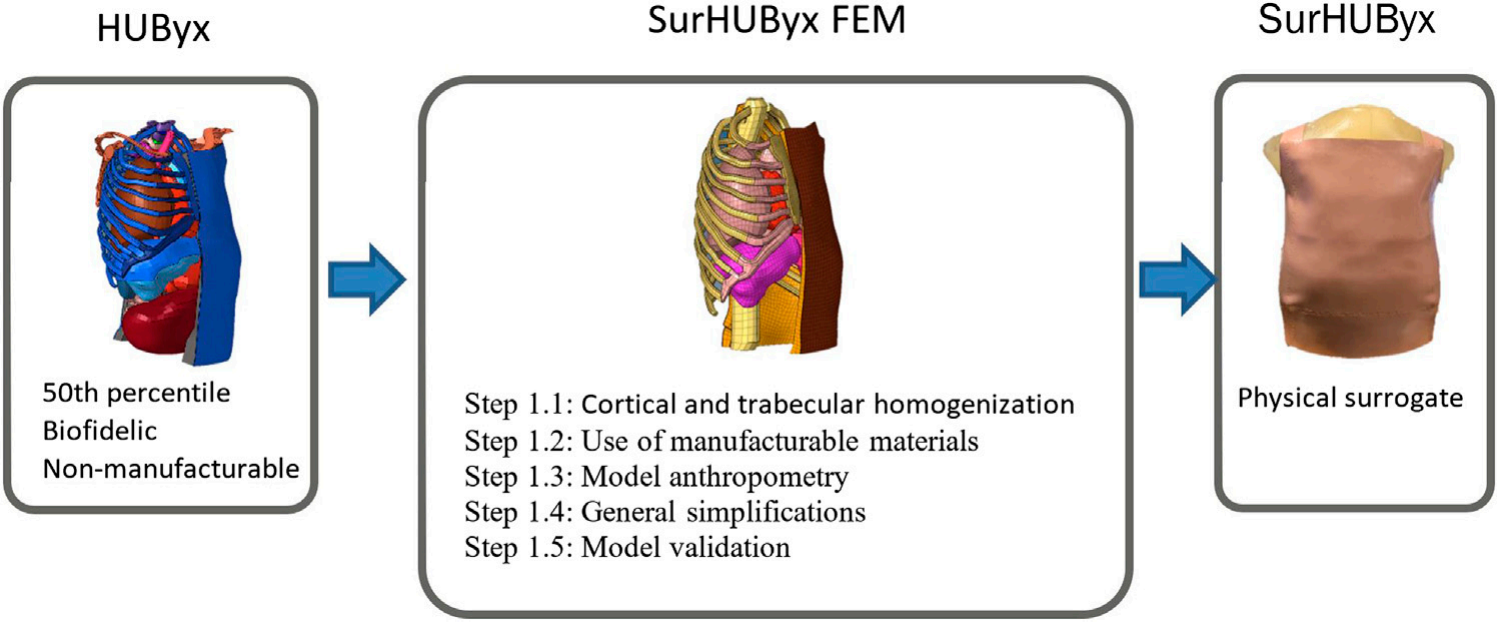
Reverse engineering procedure: from a finite element model to its physical twin.

The reverse engineering method illustrated in Figure 1 is detailed in the corresponding subsections.

### 2.1 Cortical and trabecular homogenization

**FIGURE 2:**
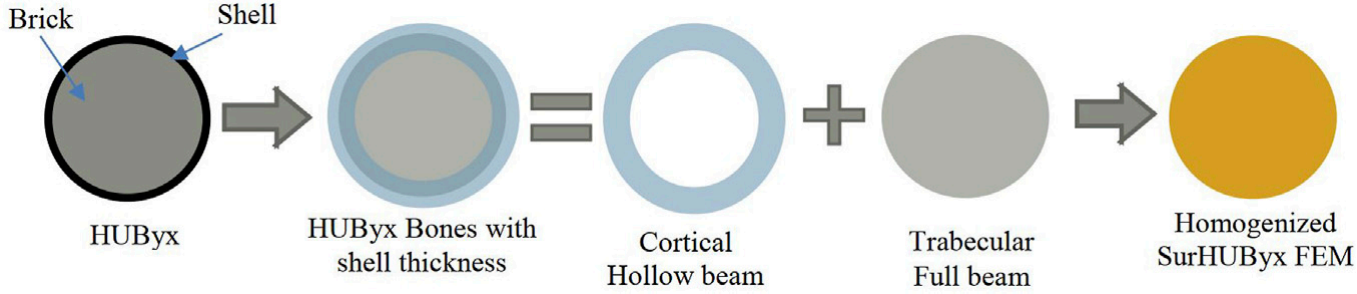
Method used for the homogenization of SurHUByx FEM bones.

HUByx bone structures were created from trabecular and cortical bone with different properties. For numerical simplification, HUByx cartilage was built with the same structure (trabecular and cortical) (Roth et al., 2013). The production of this type of structure, e.g., by 3D printing, is complex, so simplifications are required in order to create a manufacturable structure. To address this, homogenization of all bone and cartilage structures is proposed (Jianbo and Roth, 2021; Chaufer et al., 2022), as illustrated in Figure 2.

To achieve equivalence in terms of bending stiffness (EI), where E is Young’s modulus and I is the moment of inertia, a bending configuration of the homogenized structure is considered: both bones and cartilage were assumed to be circular beams loaded in a three-point bending configuration. The first step was then to calculate an equivalent homogenized modulus E_sub_ by using a mixing rule (Eq. (1)). The equivalence, in this case, can be written as Eq. 1:

$$E_{sub}=\frac{E_{cor}I_{cor}+E_{tra}I_{tra}}{I_{sub}}$$ (1)

where “sub” is the substitute parameter, “cor” is cortical, and “tra” is trabecular. Equations 2 and 3 recall the inertia of full ( $I_{f}$ ) and hollow ( $I_{h}$ ) circular beams.

$$l_{f}=\frac{\pi D^{4}}{64}$$ (2)

$$I_{h}=\frac{\pi }{64}\left(D^{4}-d^{4}\right)$$ (3)

D and d are the external and internal diameters of the circular beams, respectively. The parameters used for the cortical and trabecular materials and the new material properties used for the substitutes are given in Table 1. It should be noted that the fracture behavior of the new unified bone and cartilage has not been studied.

**TABLE 1:** Initial and new bone and costal cartilage properties.

Parameters	Initial bones Jianbo and Roth. (2021)		Initial cartilage Roth et al. (2013)		Homogenized properties	
	Trabecular	Cortical	Trabecular	Cortical	Bones	Cartilage
**Material model**	Elasto-plastic Johnson-Cook with rupture		Linear elastic		Elasto-plastic Johnson-Cook	Linear elastic
**Density (kg/mm** ^**3**^ **)**	773	1691	1000	1000	1200	1000
**Young’s modulus (MPa)**	1800	9374	50	50	20,226	148
**Fracture plastic strain**	0.03	0.02	-	-	-	-

### 2.2 Use of industry-available, manufacturable materials for the thorax

**FIGURE 3:**
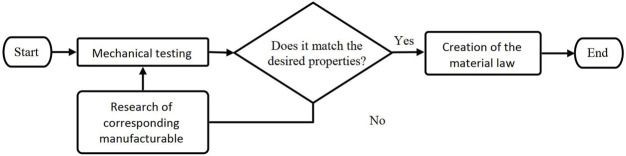
Reverse engineering procedure: from numerical properties to manufacturable materials.

The objective was to incorporate material properties of readily available, manufacturable materials into the code of the simplified FE model to accurately replicate the behavior of the human thorax and construct the physical twin of SurHUByx FEM (SurHUByx). To achieve this, a reverse engineering method was used, as detailed in Figure 3 **.** First, a manufacturable material available in the industry was mechanically tested in tension or compression, and its response was analyzed. Due to slippage issues, very soft materials were tested in compression, while harder materials were tested in tension. For compression tests, data were obtained directly from the compression machine. The local displacement of tension samples was measured using Digital Image Correlation (DIC). The repeatability of the results was ensured by using three samples of the same material. Materials were tested at two different speeds (0.02/s and 20/s) to quantify their strain rate dependence. The purpose of conducting tests at two different strain rates was to provide an estimate of the accuracy of using non-viscous material laws to model the material behavior. The experimental data were then fed into a computational code to conduct numerical tension or compression experiments to validate the numerical material law. If the response did not match these properties, another material (softer or harder) was chosen and then mechanically tested. Once the closest material was identified, its material law was implemented in the simplified FE model.

#### 2.2.1 Rib cage

Once the rib cage structure was simplified, manufacturable materials available in the industry that have similar properties as the homogenized ones had to be found to build the simplified, manufacturable FE model. To achieve this, the reverse engineering method illustrated in Figure 3 was used. After several tests, it was noticed that for the surrogate bones, industry-available manufacturable materials allowed for matching either the desired Young’s modulus or the strain to failure. As the physical surrogate is designed to help with protection assessment, bone fractures were not studied, so the strain to failure had to be slightly higher than that of human bones. Therefore, it was decided to use the manufacturable material having the most consistent strain to failure: a polyurethane resin. Since Young’s modulus for this resin was lower than the identified properties of the homogenized bones, an equivalence in terms of the moment of inertia was still used to maintain the same bending stiffness. In this step, the unknown parameter was the diameter of the ribs. As it was done in the previous section, a bending configuration of the homogenized bones was taken into consideration; the bones were assumed to be circular beams loaded in three-point bending. The equivalence can be written as follows:

$$I_{bones\_resin}=\frac{E_{bones\_sub}I_{bones\_sub}}{E_{bones\_resin}}$$ (4)

$$D_{bones\_resin}=\frac{64\times E_{bones\_sub}I_{bones\_sub}}{\pi \times E_{bones\_resin}}{}^{1/4}$$ (5)

with “bones_resin” corresponding to the parameters for the manufacturable bone resin and “bones_sub” corresponding to the computed parameters of the bone substitute.

This equivalence was done for each rib, allowing for different increases in cross-section. This entire procedure allowed us to find an industry-available, manufacturable material to build the bones of the physical surrogate: a Sika^®^ polyurethane resin. The material behavior law of this polyurethane resin was then modeled using a Johnson-Cook law and then implemented in the simplified FE model. This material was used for the ribs, spine, and sternum. The cross-section of the sternum was also increased. Since bone and cartilage are continuous, the diameter of the cartilage had to be increased as well. To find the properties of the cartilage substitute with this new geometry, an equivalence in terms of bending stiffness was used with the same hypotheses as before. The equivalence can be written as follows:

$$E_{cartilage\_resin}=\frac{E_{cartilage\_sub}I_{cartilage\_sub}}{I_{cartilage\_resin}}$$ (6)

where “cartilage_resin” corresponds to the parameters of the manufacturable cartilage resin and “cartilage_sub” corresponds to the computed parameters of the cartilage substitute.

Using Eq. 6, the new ideal cartilage properties were identified. The reverse engineering method presented in Figure 3 enabled the identification of a suitable manufacturable material, namely an elastomeric resin from Sika^®^. Hooke’s law was then employed to model the behavior of this elastomeric resin in the cartilage.

#### 2.2.2 Soft tissues

Using the same reverse engineering process as illustrated in Figure 3, materials with mechanical properties closest to the initial FE model (Roth et al., 2013) were identified for soft tissues (skin, muscle, fat, internal organs, and the mediastinum). Styrene-Ethylene-Butylene-Styrene (SEBS) based gel, which is considered to be a good substitute for human soft tissues, and has numerous advantages such as mechanical consistency and transparency (Mauzac et al., 2010; Mrozek et al., 2015; Bracq et al., 2021), was used in different concentrations for the internal organs, muscles, and mediastinum. The constitutive law of SEBS synthetic gel was implemented as a user material subroutine coded in Fortran using an Ogden model and can be expressed as follows:

$$W\left(\lambda_{1},\lambda_{2},\lambda_{3}\right)=\sum_{k=1}^{N}\frac{{µ}_{k}}{\alpha_{k}}\left(\lambda_{1}^{ak}+\lambda_{2}^{ak}+\lambda_{3}^{ak}-3\right)$$ (7)

with ${µ}_{k}$ depending on time. This visco-hyperelastic constitutive law and the corresponding mechanical parameters are clearly described in the study by Bracq et al. (Bracq et al., 2018). Bracq et al. identified the parameters of the SEBS gel used in the internal organs. As a simplification, the SEBS material behavior used in muscle and mediastinum was approximated by a linear elastic law.

Vinyl Hybrid III skin was identified as a consistent material. It was modeled using a two-parameter Ogden law (Wood Garrett et al., 2010). This material is commonly used as a biofidelic human surrogate in the automotive industry.

All material properties used in the SurHUByx FEM model are gathered in Table 2.

**TABLE 2:** Material parameters used in SurHUByx FEM.

Tissues	Bones	Cartilage	Mediastinum	Internal organs	Muscle	Skin
**Manufacturable material**	Polyurethane resin	Elastomeric resin	Gel based on SEBS	Gel based on SEBS	Gel based on SEBS	Vinyl
**Material model**	Elasto-plastic Johnson Cook	Elastic	Elastic	Ogden	Elastic	Ogden
**Density (kg/m** ^**3**^ **)**	1220	1000	1000	880	1000	1500
**Young’s modulus (MPa)**	2225	16	0.05	-	10	-
**Fracture plastic strain (-)**	0.03	-	-	-	-	-
**Yield stress (MPa)**	16.5	-	-	-	-	-
**Poisson’s ratio (-)**	0.33	0.35	0.45	0.495	0.45	0.499
**Mu 1**	-	-	-	(Time-dependent)	-	0.318
**Mu 2**	-	-	-		-	-0.401
**Alpha 1**	-	-	-	2	-	1.492
**Alpha 2**	-	-	-	-2	-	-3.316

### 2.3 Model anthropometry

The use of readily available, manufacturable industry materials in the construction of the physical surrogate required the authors to make adjustments to the bone and cartilage cross-sections. The subsequent effects of these modifications are expounded below.

#### 2.3.1 Conservation of intercostal space

The use of polyurethane resin to represent bone required the authors to increase the diameter of the ribs. This increase directly resulted in a decrease in intercostal space. Some available case reports in the open literature highlight the importance of this space (Kobayashi and Mellen, 2009). Therefore, the authors decided to maintain the same intercostal space as in the initial FE model. This resulted in an increase in thoracic height. To compare this new anthropometry with that of humans, the authors proposed to compare the rib-head positions in cadavers with the rib-head positions in the simplified FE model using the study of Mayeur et al. (Mayeur, 2013).

#### 2.3.2 Internal organ size

Since the intercostal space was preserved, the height of the simplified FE model (SurHUByx FEM) is now higher than the original FE model (HUByx). To ensure that this change in height does not induce a change in the shape of the internal organs, the authors analyzed the relationship between height and lung volume established by Hepper et al. (Hepper et al., 1960). In addition to this physiological measure, the authors used physical measurements of the lungs made by Kramer et al. (Kramer et al., 2012) to estimate the relevance of SurHUByx lung size in terms of height, width, and depth. This allowed for a comparison of organ size within natural variation. Due to the significant variability in human anatomy, other organs were scaled according to the observations made on the lungs.

### 2.4 General simplifications and model creation

Once the rib cage was homogenized and the anthropometry was compared to that of humans, the authors attempted to further simplify the initial FE model. To achieve this, a sensitivity study was conducted. Cartilage and bone were merged using the same material law, linear elastic laws were used, and the ideal Young’s modulus was sought. The mediastinum and muscle were modeled using the same material law. Organs within the mediastinum were merged. Finally, the skin was removed. In addition, the geometry of the spine was simplified: all vertebrae were merged to form a single, continuous part. Once the initial FE model was sufficiently simplified, the modeling of the SurHUByx FEM began. The SurHUByx FEM was built using Hypermesh, and calculations were conducted using the Radioss solver. As for HUByx, the interaction between the organs was ensured by modeling the mediastinum with SPH particles. All other parts were built with 8-node brick elements. In order to avoid penetration between slave and master surfaces, general contact interfaces were used between the different organs. To quantify the benefits of using the simplified SurHUByx FEM over the HUByx FE model, the authors compared the two models in terms of computational cost and the number of elements.

### 2.5 Model validation

Biomechanical corridors established by Bir et al. (Bir et al., 2004) were used to evaluate the consistency of the model. These corridors were established from tests on 13 Post Mortem Human Subjects (PMHS) impacted over the sternum by different rigid projectiles at different velocities. Three impact cases were conducted: Case A with a 140 g projectile fired at 20 m/s, Case B (140g – 40 m/s), and Case C (30g – 60 m/s). In order to have quantitative data to compare within these biomechanical corridors, numerical replications of these physical tests were performed: an initial velocity was applied to the FE model of the impactor that impacted the SurHUByx FEM in a manner similar to the experimental tests. Finally, a comparison was made between the experimental and numerical thorax responses in terms of force-time and deflection-time curves and Vc_max_ values over the three impact conditions.

## 3 Results

### 3.1 Anthropometry

**FIGURE 4:**
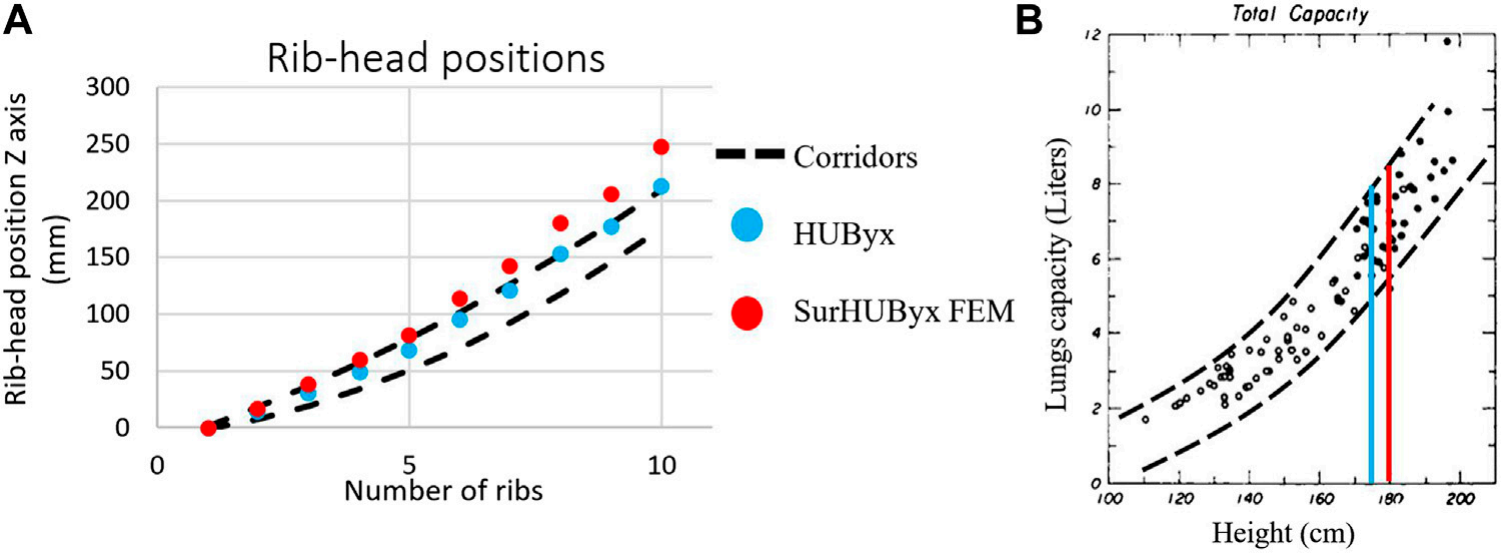
Comparison of rib-head positions of HUByx and SurHUBx within experimental corridors [28] **(A)** Relationship between height and lung volume [29] **(B)**.

Figure 4 shows a comparison between the corridors created by Mayeur (Mayeur, 2013) from measurements of rib-head positions in 19 subjects with HUByx and SurHUByx FEM. The HUByx rib-head positions were in the upper part of the corridors. SurHUByx FEM rib-head positions were slightly outside the corridors.

As the height of the rib cage was increased in SurHUByx FEM, it was verified that the size of the internal organs of HUByx still corresponded to this anthropometry or if a modification was required before implementing these organs into SurHUByx FEM. For this purpose, the relationship between the height of the subjects and the lung volume established by Hepper et al. (Hepper et al., 1960) was used (illustrated in Figure 4B). It was shown that for the same height, the total lung capacity can vary by up to 20% between subjects. In addition, the study conducted by Kramer et al. (Kramer et al., 2012) demonstrated that the lung size in the SurHUByx model was within the average range of values measured in 81 human male adults, as presented in Table 3. By extension, other organs were used as such, so SurHUByx has the exact same lungs, heart, liver, and spleen as HUByx.

**TABLE 3:** Comparison of linear dimensions between SurHUByx and a human data set.

	Lung	Peak-to-peak height (cm)	Width (cm)	Depth (cm)
Human data set	Right	21.0 ± 2.1	12.3 ± 1.1	18.0 ± 1.15
	Left	21.0 ± 2.1	12.3 ± 1.1	18.0 ± 1.15
SurHUByx	Right	20.8	10.5	17.1
	Left	19.4	11.5	17.3

### 3.2 Simplifications

The sensitivity study showed that the skin was necessary to contain the muscle. It was not appropriate to use the same material for cartilage and bone, but this did highlight the efficiency of the dampening effect provided by the costal cartilage and proved the importance of building a rib cage out of two different materials. The mediastinum and the muscle also have to be modeled using different materials. Finally, only the major internal organs (heart, lungs, liver, and spleen) were represented. The others were merged with the mediastinum. As a simplification, the fat and muscle components were merged together, resulting in a softer overall muscle material in the physical surrogate. This simplification was done in order to improve the manufacturability of the surrogate model.

As a result, the SurHUByx FEM is composed of the mediastinum, the lungs, the heart, the liver, the spleen, the ribs, the costal cartilage, the sternum, the spine, the muscles, and the skin. Finally, the SurHUByx FEM consists of 37,000 8-node brick elements.

### 3.3 Model validation

**FIGURE 5:**
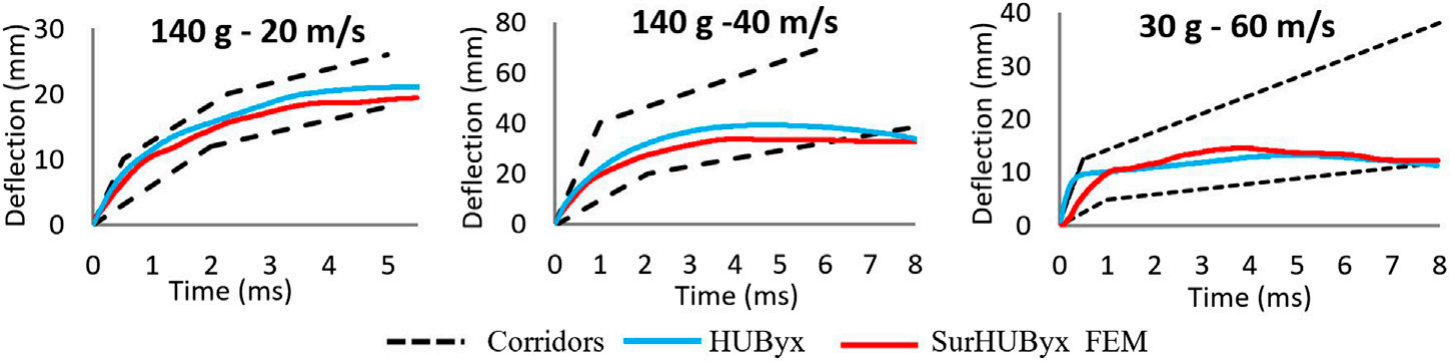
Deflection time curves for the three cases of Bir impact.

**FIGURE 6:**
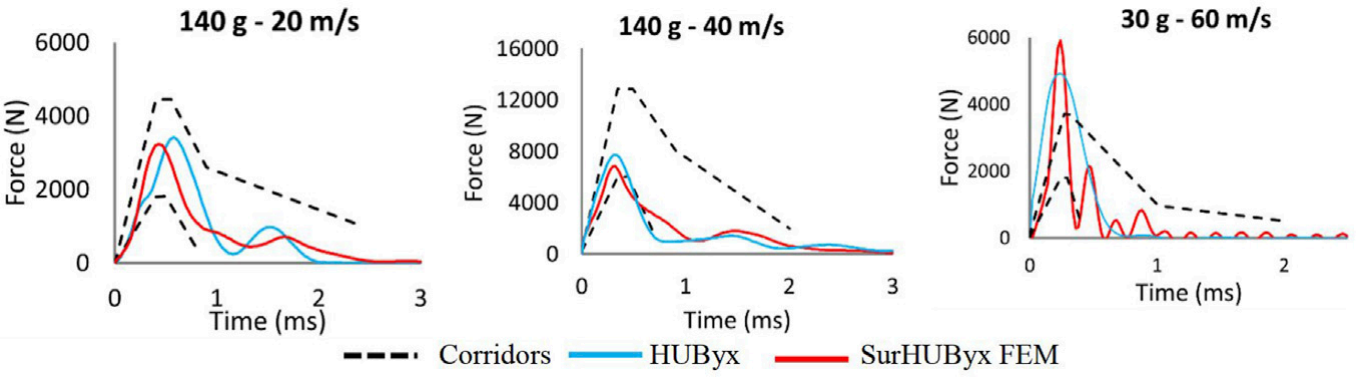
Force time curves for the three impact cases of Bir impacts.

**FIGURE 7:**
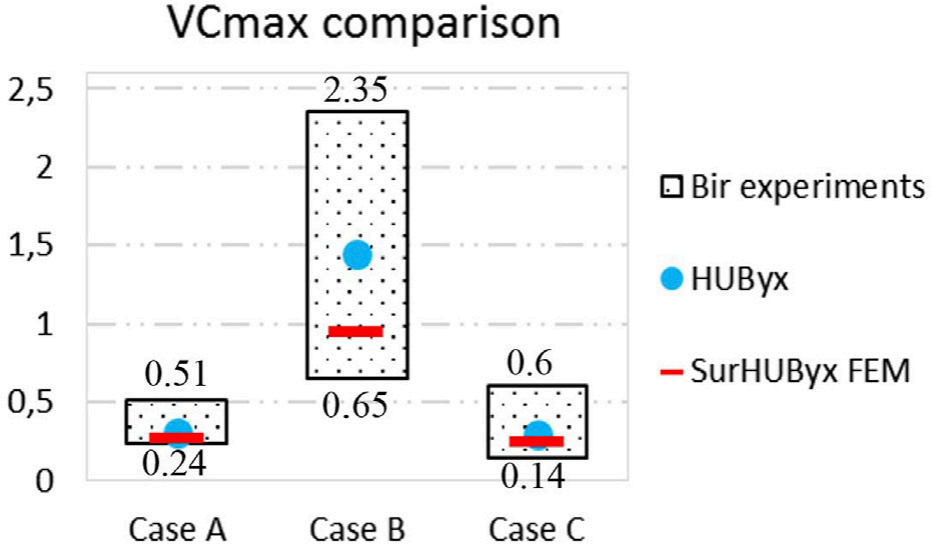
VCmax comparisons between cadaver experiments, HUByx and SurHUByx FEM.

Figure 5 shows the different deflection-time histories in relation to their corresponding experimental corridors for impacts A, B, and C. Figure 6 shows the different force-time histories in relation to their corresponding experimental corridors for impacts A, B, and C. The SurHUByx FEM with its new material properties of manufacturable materials showed consistent behavior with the Bir et al. corridors for both deflection and force-time curves. In addition to the numerical deflection measurements, the typical parameter for thoracic impacts, VC_max_ (Maximal Viscous Criterion), was also calculated for the three impact conditions. Figure 7 illustrates the SurHUByx FEM, HUByx, and experimental range VC_max_ values. For all three impact conditions, the VC_max_ obtained with SurHUByx FEM was consistent with cadaveric experiments.

#### 3.3.1 Global gain

The results presented in the previous sections proved the efficiency of the SurHUByx FEM in terms of mechanical response. Since the results were close to those obtained with HUByx, the authors proposed comparing the two FE models in terms of numerical performance. Table 4 shows the quantitative differences between these two models. As bones and cartilage were homogenized in SurHUByx FEM, no shell elements were required. Even if some organs were removed, the number of brick elements increased slightly due to the modeling of the spine, which was modeled with shell elements in HUByx. The number of SPH particles in the simplified FE model was divided by four. Finally, the computation time was reduced by an average of 40% with SurHUByx FEM.

**TABLE 4:** Vc_max_ comparisons between cadaveric experiments, HUByx and SurHUByx: Numerical comparison between HUByx and SurHUByx FEM.

	HUByx	SurHUByx FEM
Number of shell elements	77,800	0
Number of brick elements	36,600	37,000
Number of SPH particles	109,000	26,265
CPU time for Case A (6 m)	13 h	10 h
CPU time for Case B (10 m)	15 h 20 min	7 h 45 min
CPU time for Case C (8 m)	12 h 30	6 h 45

## 4 Discussion

In mechanics, numerical models are often used to reproduce physical phenomena (Giglio et al., 2012; Kanehira et al., 2020). The classical approach consists of modeling a physical mechanism in order to predict its behavior using numerical simulations. Once validated, the numerical model is used to replace physical experiments in preliminary studies, but physical experiments are still needed for validation. This study proposes a reverse engineering method by creating a numerical model that will be used to select materials that are consistent with the desired behavior. Once the model is validated, it will be the basis for its physical twin. This procedure allows material parameters to be easily varied to see their influence and helps choose manufacturable materials to use in building the physical model. A similar procedure was used by Roberts et al. in the development of HSTM and HTFEM (Roberts et al., 2005b; Roberts et al., 2007b). The limitation of HSTM and HTFEM is that they have not been validated against either animal or cadaveric experimental data. To go further, this study used as a reference a biofidelic finite element model named HUByx, which represents the 50th percentile human thorax. Since it was done in a biomechanical framework to account for the wide variability of human morphologies and human responses under load, biomechanical corridors were used for validation purposes. It is generally accepted that the numerical response of a model must be within the experimental corridors to be validated.

In this study, the new simplified FEM anthropometry was compared to experimental data, allowing us to ensure that the SurHUByx FEM was consistent with the 50th percentile. The rib-head position corridor established by Mayeur et al. (Mayeur, 2013) was used. HUByx, which was in the 50th percentile, was in the upper part of the corridor, while SurHUByx FEM was slightly outside of it. The subjects used by Mayeur were all in the 50th percentile, but all of them were in the small 50th percentile, with an average height of 170 cm (160 cm for the smallest and 178 cm for the tallest). Thus, this corridor corresponds to a part of the 50th percentile. Regarding the width of the corridor obtained from the measurements of rib-head position on 19 subjects in the lower 50th percentile, SurHUByx FEM rib-head positions that were slightly outside the corridors can be assimilated to the 50th percentile. This increase in height was accounted for in the VC_max_ evaluation by using the real thoracic depth in the calculation. The effect of geometric size on the injury criteria of Bir et al. was estimated in (Roth et al., 2013) by scaling the HUByx model (50th percentile) from the 5th percentile to the 95th percentile. The results showed that the behavior of each model was within the corridors. Thus, reasonable geometric modifications of the original model still led to validated models, as very few differences were observed in terms of numerical response (Roth et al., 2013). These observations allowed the authors to slightly modify the HUByx model to build the SurHUByx FEM.

Then, the relationship of lung volume to the height of the subject was used (Hepper et al., 1960). It was found that for the same height, total lung capacity can vary up to 20% between subjects. Furthermore, a comparison of SurHUByx lung size with the measurements made by Kramer et al. (Kramer et al., 2012) proved that SurHUByx can have exactly the same lungs as HUByx. In the study by Shen et al. (Shen et al., 2008), a sensitivity analysis was conducted to evaluate the effect of lung material properties on impact duration, deformation, and lung pressure. The results showed that lung material properties had a negligible effect on impact duration and deformation but a significant effect on lung pressure. Therefore, variations in the amount of lung material behind the sternum, which could result in smaller or larger lungs, would have little effect on the accuracy of the model in terms of global force and displacement.

As SurHUByx FEM is in the 50th percentile, it can be compared to the corridors established by Bir et al. (Bir et al., 2004). The simplified model was validated against the experimental corridors established by Bir et al. for impacts on the thorax, and the results showed that the SurHUByx FEM had a consistent mechanical response with the experimental data. These results showed that both the initial and the simplified FEM were within the biomechanical corridors. Since the behavior of each human is different, corridors are used in biomechanics, so no conclusion or observation can be drawn concerning whether SurHUByx or HUByx have a closer dynamic response to the human body.

Additionally, the SurHUByx FEM had a computational cost advantage over the HUByx FE model, making it more efficient for numerical simulations. However, it should be noted that the validation was performed using only global parameters, and no conclusions can be drawn about the model’s ability to predict local parameters or damage metrics. Thus, SurHUByx FEM can be used as a preliminary study to gain CPU time, but for more accurate results, the HUByx FEM model can be used (Bodo et al., 2017; Bracq et al., 2019b). Consequently, SurHUByx and HUByx can be seen as two twins with external similarities but different dynamic responses. With SurHUByx FEM, the computation time and manufacturing ability were optimized.

Finally, the validation proposed in this study is based only on cadaver experiments. Experiments conducted on live animals are another way to evaluate surrogate performance. These two methods seem to be complementary: PMHS offers the best morphological similarities (Bir et al., 2004), and pigs have the best pathophysiological similarities (Prat et al., 2010). A study comparing PMHS and porcine experiments regarding ballistic impacts exists (Prat et al., 2012). To the best of the authors’ knowledge, no study has compared the behavior of PMHS and pigs with that of living humans in a ballistic context.

Even though this new FE model has proven its biofidelity and its savings in terms of CPU time, it cannot be used to study bone fractures. In fact, the authors prioritized the replication of the chest wall motion. The accurate chest wall motion on the surrogate would help to study the dynamic backface deformation of the armor, which is not possible with the actual standard such as clay, which only measures the residual backface deformation. Such a surrogate would go further than actual surrogates that allow observation of dynamic backface deformation, such as the SEBS gel-based block (Mauzac et al., 2010), by allowing measurement of internal pressure within organs and by capturing multiple data points within the surrogate. Future work is needed on the SurHUByx FEM to predict injury to the specified organs. Local criteria can be used, as already done by Taddei et al. (Taddei et al., 2021).

**FIGURE 8:**
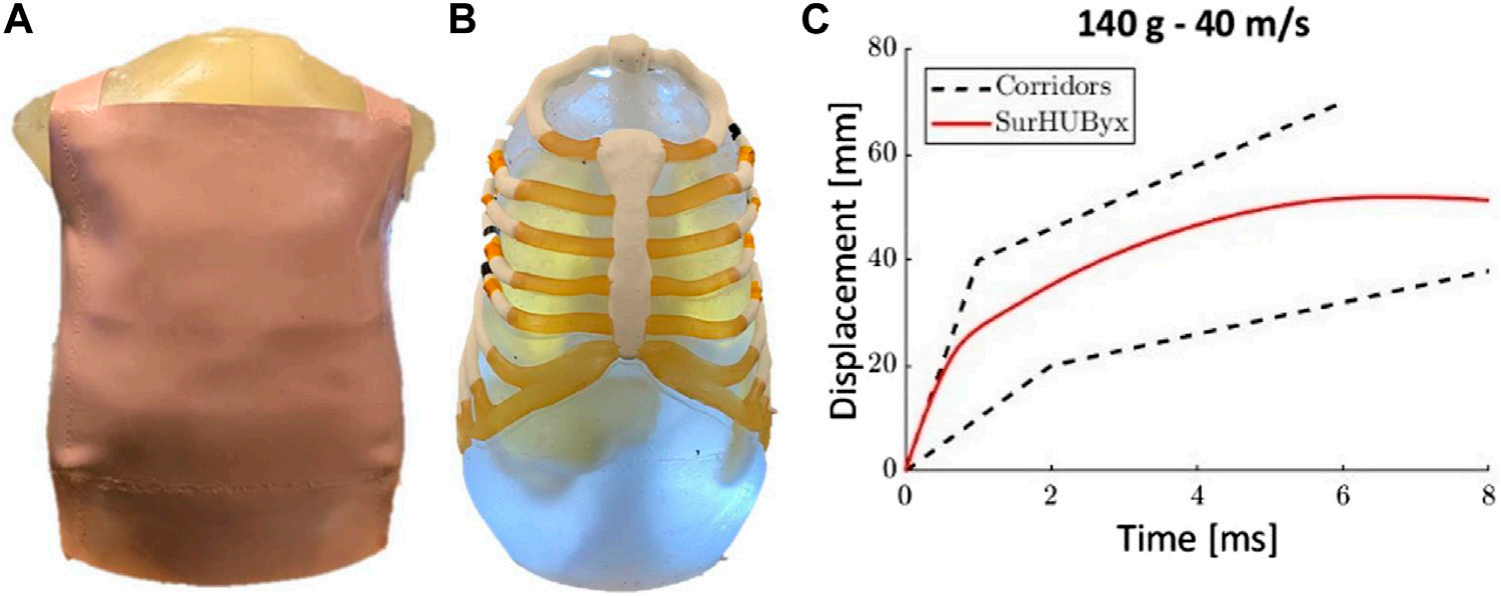
SurHUByx external view **(A)**, SurHUByx mediastinal and thoracic view **(B)** and SurHubyx displacement time curve for case B of the Bir experiments **(C)**.

This study was the first step in the design of SurHUByx, the physical twin of SurHUByx FEM. This reverse engineering procedure allowed for the transition from a numerical framework to a physical one, with the step of physically recreating the experiments of Bir et al. As a result, a physical surrogate based on the SurHUByx FEM can be built using manufacturable materials. These surrogate manufacturable materials can also be seen as a limitation. For example, the vinyl material used for the skin has different properties than human skin. However, it was chosen because it was readily available and its material properties are documented in the open literature (Wood Garrett et al., 2010). Replication cases are currently in progress with the physical SurHUByx, and they provide very promising results for evaluating body armor in physical contexts (Figure 8). These results validate the reverse engineering procedure used in this study. Once validated, field impact cases could be replicated on the SurHUByx to link data collected by embedded sensors in the thorax to injury scales for each organ. These capabilities would allow for the evaluation of different body armor systems. Further research could include identifying different field impact cases to improve wound and injury prediction using these functions. Additionally, future research could focus on finding a way to measure the VC response without altering the behavior of the surrogate. Finally, similar work could be done to develop other anthropometric surrogates.

## 5 Conclusion

A simplified version of an existing biofidelic finite element model of the human thorax, called SurHUByx FEM, was created by simplifying the structure of the HUByx model. Reverse engineering methods were used to find industry-available, manufacturable materials with consistent properties. The properties of these materials necessitated changes to the rib cross-sections to achieve equivalence in terms of bending stiffness. To preserve intercostal space, the size of the rib cage was increased. Comparison with experimental data showed that SurHUByx FEM was still consistent with a 50th percentile human. This new model was then validated using experimental data in a ballistic impact context and was found to behave consistently with biomechanical corridors established from cadaveric experiments. Moreover, the use of SurHUByx FEM halved the CPU time. This validation process was a successful first step in the design of SurHUByx: a physical version of the simplified model.

## Data Availability

The original contributions presented in the study are included in the article/supplementary material, further inquiries can be directed to the corresponding authors.
